# Associations among 25-year trends in diet, cholesterol and BMI from 140,000 observations in men and women in Northern Sweden

**DOI:** 10.1186/1475-2891-11-40

**Published:** 2012-06-11

**Authors:** Ingegerd Johansson, Lena Maria Nilsson, Birgitta Stegmayr, Kurt Boman, Göran Hallmans, Anna Winkvist

**Affiliations:** 1 Department of Odontology, Umeå University, 901 87, Umeå, Sweden; 2 Department of Public Health and Clinical Medicine section of nutrition, Umeå University, Umeå, Sweden; 3 The National Board of Welfare, Stockholm, Sweden; 4 Department of Medicine, Skellefteå County Hospital, Skellefteå, Sweden; 5 Department of Internal Medicine and Clinical Nutrition, The Sahlgrenska Academy, University of Gothenburg, Göteborg, Sweden

**Keywords:** Diet, Fat, Alcohol, Cholesterol, BMI, Tme trend, Sweden

## Abstract

**Background:**

In the 1970s, men in northern Sweden had among the highest prevalences of cardiovascular diseases (CVD) worldwide. An intervention program combining population- and individual-oriented activities was initiated in 1985. Concurrently, collection of information on medical risk factors, lifestyle and anthropometry started. Today, these data make up one of the largest databases in the world on diet intake in a population-based sample, both in terms of sample size and follow-up period. The study examines trends in food and nutrient intake, serum cholesterol and body mass index (BMI) from 1986 to 2010 in northern Sweden.

**Methods:**

Cross-sectional information on self-reported food and nutrient intake and measured body weight, height, and serum cholesterol were compiled for over 140,000 observations. Trends and trend breaks over the 25-year period were evaluated for energy-providing nutrients, foods contributing to fat intake, serum cholesterol and BMI.

**Results:**

Reported intake of fat exhibited two significant trend breaks in both sexes: a decrease between 1986 and 1992 and an increase from 2002 (women) or 2004 (men). A reverse trend was noted for carbohydrates, whereas protein intake remained unchanged during the 25-year period. Significant trend breaks in intake of foods contributing to total fat intake were seen. Reported intake of wine increased sharply for both sexes (more so for women) and export beer increased for men. BMI increased continuously for both sexes, whereas serum cholesterol levels decreased during 1986 - 2004, remained unchanged until 2007 and then began to rise. The increase in serum cholesterol coincided with the increase in fat intake, especially with intake of saturated fat and fats for spreading on bread and cooking.

**Conclusions:**

Men and women in northern Sweden decreased their reported fat intake in the first 7 years (1986–1992) of an intervention program. After 2004 fat intake increased sharply for both genders, which coincided with introduction of a positive media support for low carbohydrate-high-fat (LCHF) diet. The decrease and following increase in cholesterol levels occurred simultaneously with the time trends in food selection, whereas a constant increase in BMI remained unaltered. These changes in risk factors may have important effects on primary and secondary prevention of cardiovascular disease (CVD).

## Introduction

In the 1970s it was noted that the prevalence of cardiovascular diseases (CVD) was higher in the Västerbotten area in northern Sweden than elsewhere in the country [1]. In fact, it was among the highest among men in the world [2]. Therefore, in 1985 a community-based prevention programme was initiated in the municipality of Norsjö, Västerbotten county. The program was later extended to the entire county [3]. The Västerbotten Intervention Programme (VIP), which still runs, then combined population-oriented activities, such as a food labelling system, health information meetings, cooking demonstrations, and individual-oriented activities, such as offering health examinations and counselling to inhabitants when they turned 30, 40, 50 and 60 years of age [4,5]. Intervention on diet was a central component throughout the program. Concurrently, Västerbotten and Norrbotten, the two northern-most counties of Sweden, joined the WHO MONICA Project (Multinational Monitoring of Trends and Determinants in Cardiovascular Diseases) [6]. Data collection within VIP and MONICA was harmonized to allow for comparisons and collaborative evaluations.

Changes in lifestyle factors during 1986 – 1999 have been reported from the MONICA surveys [7-9]. It was noted that the use of butter and high-fat milk declined in favour of low-fat margarine and low-fat milk products. Among both men and women, the intake of alcoholic beverages increased. No change in leisure-time physical activity was noted. Smoking declined, but use of snuff increased. During the same period, decreasing numbers of fatal and non-fatal strokes and myocardial infarctions were reported for the region as monitored by the Northern Sweden MONICA study [10,11]. The association between nutrition and health is complex. It involves specific food components, interactions among those food components, and interactions with genetic factors and individual needs. Early on, the ecological Seven Countries’ Study claimed that a substantial proportion of the regional variation in CVD mortality was explained by differences in intake of saturated and monounsaturated fatty acids, and they pointed out that mean levels of total cholesterol correlated with mean intakes of saturated fatty acids [12,13]. However, a recent review on the role of fats and fatty acids on human health concluded that the relationship is more complex [14]. Trans fatty acids increase the risk, fish or n-3 long-chain polyunsaturated fats decrease the risk, but the data are conflicting or insufficient to convict or free total fat intake or other fat fractions with respect to CVD risk. Thus, further research is needed, especially focusing on long-term dietary intake.

Data from the Västerbotten Intervention Programme and the Northern Sweden MONICA study together form one of the largest diet databases in the world with the longest follow up time on diet intake in a population-based sample. It has been used for several nested case referent and cohort studies (e.g. [15-17]), ecological studies in the European Prospective Investigation into Cancer and Nutrition (EPIC) and other consortia (e.g. [18-20]), and nutrient-gene interaction studies [20]. Here, we evaluate trends in food intake, serum cholesterol and BMI in people living in northern Sweden over the 25-year period covered by the database. This evaluation thus encompasses periods of active intervention on diet and lifestyle and a period of increasing popularity and mass media reports on the benefits of very-low-carbohydrate, high-fat diets (LCHF) [21]. A main focus of this explorative study was to evaluate if food selections, fat, carbohydrate and protein intakes had stabilized at the improved levels seen during the 1990s [7], and adhere to the recommendations given by the Nordic Nutrition Recommendations or to the increasing media support for high-fat, low-carbohydrate diets that have received much coverage in recent years.

## Methods

### Study cohort

The present study is based on the Northern Sweden Diet database, which is formed by information from the participants in two population-based studies in Northern Sweden, i.e.*,* the Västerbotten Intervention Programme (VIP) and the Northern Sweden MONICA study. Both studies had their first survey in 1986, and highly similar health survey procedures are used. At present, data through 2010 are included. All participants provided written informed consent, and the Regional Ethical Review Board of Northern Sweden approved both basic studies and the present study.

### The Västerbotten intervention programme (VIP)

In VIP residents in the county of Västerbotten are invited to participate in a health survey at their local primary health care centre when they turn 40, 50 or 60 years [5,22]. Prior to 1994, residents 30 years of age were also invited, and some primary health care centres continue to invite this age group.

Participants were asked to fast for at least four hours prior to the health survey visit. At the visit a diet and lifestyle questionnaire was answered, body measures were taken, including weight and height, and blood was drawn. Participation rate has varied; it was 48–57% from 1991 to 1995 and thereafter increased to around 70% from 2005 and onwards**.** The lower participation rates in the earlier years mainly reflect low rates among 30-year olds, who primarily were invited in the early years. Differences in social characteristics and CVD risk factors between VIP participants and regional population samples from a Population and Housing Census screening [23] and the randomly selected MONICA participants were marginal [24].

### The Northern Sweden MONICA project

In the MONICA project independent, cross-sectional samples are randomly selected from updated population registers for inhabitants in the counties of Västerbotten and Norrbotten every 4th to 5th year [6]. Sampling is stratified to include equal numbers by gender and 10-year age groups (25–65 year olds in the 1986 and 1990 surveys, thereafter the age range was 25–75 years). The present study involves survey data from 1986, 1990, 1994, 1999, 2004 and 2009, and recall visits in 1999 of participants from 1986, 1990 and 1994. Participation rates have varied between 69% and 81% by survey occasion. Survey procedures were as described for VIP, except for 1990 when a shortened diet questionnaire was used. Minimal evidence of systematic differences in socio-demographic characteristics for participants over time, or between participants and non-participants, has been found [25,26].

### Food intake measurement

A semi-quantitative food frequency questionnaire (the Northern Sweden Food Frequency Questionnaire (FFQ) was used to estimate dietary intake in both the VIP and MONICA projects [27]. In 1996, the originally 84-question questionnaire was reduced to a 64-question version in VIP for financial reasons. However, intake frequencies were reported on an identical nine-level scale, and most questions in the 64 FFQ-version were kept identical to that of the longer version. From 1992 the FFQ became optically readable. In the present study only sections of foods, where the questions were identical in the two different versions, were studied. Four photos with increasing amounts of meat, vegetables and potato/rice/pasta were used to indicate portion sizes of these types of foods. For other foods either gender and age-specific portion sizes or fixed sizes, such as for an apple or an egg, were used [27].

Reported intake frequencies were transformed into number of intakes per day, and energy and nutrient intake was estimated by weighting reported intake frequencies with portion sizes and nutrient content from the database at the National Food Administration, Sweden (http://www.slv.se) as described earlier [27].

The Northern Sweden FFQ has been validated for several nutrients, including energy, fat, protein, carbohydrate, fatty acids, phytosterols, vitamin and mineral estimates, against repeated 24-hour dietary records (24HR) and/or biological markers [27-31]. The FFQ had high reproducibility, and in comparison with 24HR, acceptable ranking capacity for energy and several nutrients, i.e. correlation coefficients above 0.50 and intake ratios close to 1. Like other dietary recording methods, underreporting in relation to estimated basal metabolism was frequent [31]. Further, gender and age group stratified ranking of individuals into quintiles has been found similar when based on the distributions of energy and nutrient intake estimated by the longer and shorter FFQ version [29].

### Alcohol intake

For the present study three FFQ questions that were identical over the entire study period were used, i.e. intake frequency of 3.5% alcohol (export) bear, wine and spirits.

### Exclusion criteria for diet records, handling of missing values, and final study cohort for diet estimates

**Figure 1:**
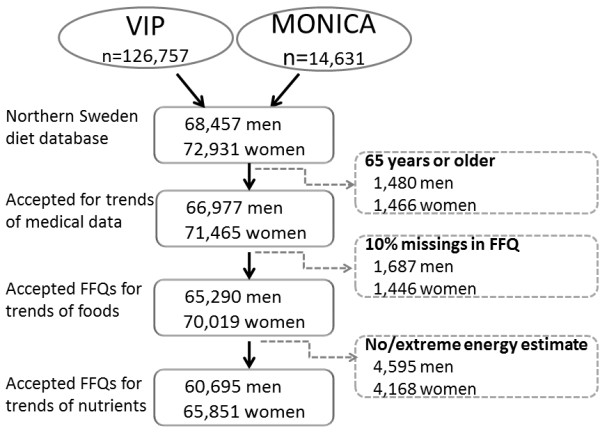
**Number of observations in the study cohort.** Number of observations excluded by various criteria and observations remaining for analyses are given.

In August 2010, the VIP cohort included food intake information from 126,757 observations and the MONICA cohort information from 14,631 observations rendering 141,388 observations in the Northern Sweden Diet database for the period 1986 to 2010 (Figure 1). Of these 25% had participated twice and <0.1% three times. The present study employed data from all observations, after exclusion of 2,946 observations for participants older than 65 years, as being beyond the original inclusion age span (Figure 1). The longer FFQ version had been used in 35% and the shorter version in 65% of the employed observations.

Observations that had 10% or more unanswered FFQ questions were excluded from all analyses on diet intake (n = 3,133). Observations where any of the three portion-size questions was unanswered and observations with extreme ratios between reported caloric intake and estimated basal metabolic rate (food intake level, FIL; [32] were also excluded (n = 8,719). Thus, observations with FIL values below the 1^st^ or above the 99^th^ percentile values, and subjects lacking information on body weight needed for FIL calculation were excluded. This left a final study cohort for analysis of trends in nutrient intake of 126,590 observations (60,770 men and 65,820 women), and in foods/food aggregates of 135,309 observations (65,290 men and 70,019 women), and in medical data of 138,442 observations (66,977 men and 71,465 women) (Figure 1).

### Body mass index and cholesterol measures

Body weight and height were measured when the participant wore light clothes, but no shoes. Measurements were done by trained nurses by use of standardized weight and measuring scales. BMI was calculated as weight in kg/height in m^2^. A BMI could not be calculated for 0.6% of the observations due to missing height or weight. BMI values below 10 (23 observations) and above 100 (3 observations), and one recording of a height of 270 cm were excluded. In the final group the 1^st^ to 99^th^ percentile values for BMI ranged from 18.8 to 39.2, height from 152 to 192 cm and body weight from 50 to 118 kg.

Venous blood samples were drawn without stasis into evacuated glass tubes after at least 4 hours of fasting. In VIP, cholesterol in serum was measured by a Reflotron bench top analyser (Boehringer Mannheim GmbH Diagnostica, Germany) at the health centre until 2009, and thereafter by an enzymatic routine method at the clinical chemistry department at the nearest hospital. The Reflotron measures have been validated and calibrated relative to the enzymatic measures of cholesterol. The mean difference between the two methods has been reported to be 0.04 mmol/L [33], and an algorithm for calibrating enzymatic measures to Refloton measures has been calculated (cholesterol_enzymtic method_ =0.738 + (0.901*cholesterol_reflotron method_; personal communication Stenlund H, Department of Epidemiology, Umeå University). Cholesterol data from 2008 and earlier were transformed according to this algorithm to match later enzymatic measures. In MONICA cholesterol was measured with the enzymatic method at all surveys. Serum cholesterol values were missing for 5.7% of the observations accepted for medical evaluations.

### Statistical analyses

Data analyses were performed with SAS version 9.2 (SAS Institute Inc., Cary, NC, USA). All statistical analyses were performed separately for men and women in 10-year age strata or combined. Frequencies of food intakes and alcohol-containing drinks were transformed into intakes per week. Estimated total fat, protein and carbohydrate intakes were standardized for reported energy intake by dividing the energy from the nutrient by total energy in per cent, i.e. fatE%, proteinE%, and carbohydrateE%. BMI was categorized into three levels (<20, 20-27, and >27), which were used for BMI adjustment. For descriptive purposes, means and measures of variation standardized for age, and for dietary data also BMI, were calculated by the GLM procedure in SAS.

Multivariate partial least square (PLS; SIMCA P+, version 12.0, Umetrics AB, Umeå, Sweden) regression was used to identify foods that contributed significantly to high-fat intake and high-cholesterol levels in the Northern Sweden population. PLS was not used to search for foods associated with BMI because of the well-known misreporting bias in relation to BMI [34]. In contrast to traditional regression models, PLS modelling is suitable for data where the x variables co-vary, such as food selections. Dietary variables, age and BMI built the X block, and reported total fat intake (E%) or total serum cholesterol level, built the Y block (outcomes). Variables were autoscaled to unit variance, and cross-validated prediction of Y was calculated [35]. Cross validation was done by a systematic prediction of one 7th of the data by the remaining 6/7^th^ of the data. The importance of each x variable in explaining the variation in y is displayed as PLS loading correlations. R^2^ and Q^2^ values give the capacity of the x-variables to explain (R^2^) and predict (Q^2^) the outcome.

Trends and trend breaks were tested using the Joinpoint Regression programme from the National Cancer Institute, USA (http://www.surveillance.cancer.gov/joinpoint). Joinpoint is a regression programme, which connects lines together at “joinpoints” by taking trend data and fitting the simplest joinpoint that the data allow; it also tests if a change in the trend is statistically significant [36]. The test of significance uses a Monte Carlo Permutation method.

Statistical tests were two-sided and p-values <0.05 were considered statistically significant.

## Results

The number of observations per year that were acceptable for analysis of trends in food intake ranged from 3,292 to 9,883, except for 1986 and 1990 when only 1,515 and 1,549 observations, respectively, were available. The proportion of women ranged between 49.4% and 53.7% in the various study years. Due to the removal of a mandatory invitation to 30-year olds in VIP, the numbers for 30-year-olds in 2000, 2001, 2003, 2006, 2007, 2008 and 2010 ranged between 38 and 70 observations, but in other years the numbers ranged from 310 to 1,861. The 30-year-olds were kept in the data analyses as they were within the original inclusion age range. Filled symbols (means) in figures indicate years where the numbers exceed or are equal to 310. The numbers of observations per year used for nutrient intake evaluations were slightly fewer than those for food items in each year for both genders and all 10-year age groups.

### Changing intake patterns for fat and carbohydrate 1986 to 2010

**Figure 2:**
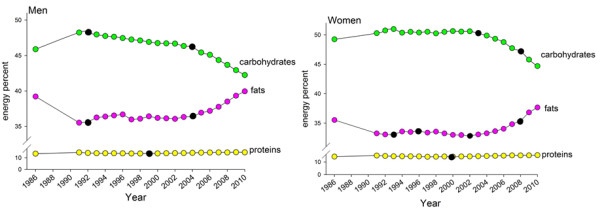
**Mean intake of fat, protein and carbohydrate expressed as energy from each nutrient in per cent of total energy intake.** Mean intakes, adjusted for age and BMI for the period 1986 to 2010 for men and women. Information was not available for 1987 to 1990 since the VIP FFQ was not fully harmonized until 1991, and the reduced FFQ version used in MONICA year 1990 was not acceptable for nutrient estimation. Black dots (●) show years with a trend shift as indicated by the Joint Point statistical programme.

In 1986, mean reported fat intake, adjusted for age and BMI, was 39.2% of the men’s total reported energy intake (E%). Carbohydrates made up 45.9 E% and protein 13.6 E%. For women the corresponding levels were 35.5 E%, 49.2 E% and 14.3 E% for fat, carbohydrates and protein, respectively, in the same year (Figrue 2). From 1986 to 2010 two significant trend breaks in reported fat intake were identified for both men and women. A decrease from 1986 to 1992 resulted in a reduction of reported fat intake by 2.9 E% in men and 4.4 E% in women. These levels stayed largely stable until 2002–2004, and thereafter a significant increase occurred, and reported fat intake reached levels above those in 1986. Thus, in 2010 men got 39.9% and women 37.7% of their reported total energy intake from fat. The trends were similar in 10-year age groups (Additional file 1: Figure S1). Intake of saturated fat correlated highly with total fat, i.e. Spearman correlation coefficients were 0.86 for men and 0.87 for women. Reported intake of saturated fat followed a similar pattern as total fat, and the correlation coefficients were virtually identical each year (data not shown). In the same period reciprocal trend shifts were seen for reported carbohydrate intake (Figure 2). The fraction of energy originating from protein was virtually unchanged over the 25-year period, i.e. a slight increase by 0.9 E% was seen for both men and women but no significant trend break was identified (Figure 2).

**Time trends for foods associated with high fat intake** Fats used for spreading on bread and cooking, dairy products, oil for salad dressing or cooking, various types of meats and sausages as main dish or on sandwiches, pizza, deep- fried potato chips (French fries), and non-sweet snacks (including potato and maize crisps (chips), cheese-flavoured puffed products, popcorn, and peanuts) were identified to be associated with high fat intake (fatE%) in the study population by PLS. These foods were analysed for trends in intake from 1986 to 2010 for items where the question for the specific food/food aggregate had remained identical over the entire 25-year period.

**Figure 3:**
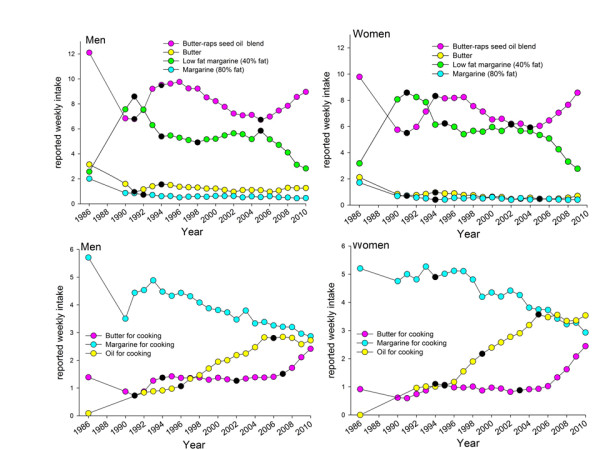
**Reported mean intake of various types of fats used for spreading on bread or cooking.** Reported weekly mean intake frequencies, adjusted for age and BMI for the period 1986 to 2010 for men and women. Information was not available for 1987 and 1988 since the VIP FFQ was not fully harmonized until 1991. Black dots (●) show years with a trend shift as indicated by the Joint Point statistical programme.

**Figure 4:**
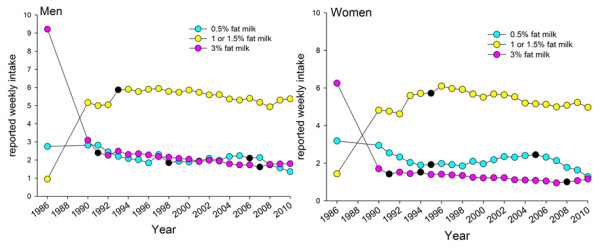
**Reported mean intake of various types of non-fermented milk.** Reported weekly mean intake frequencies, adjusted for age and BMI for the period 1986 to 2010 for men and women. Information was not available for 1987 and 1988 since the VIP FFQ was not fully harmonized until 1991. Black dots (●) show years with a trend shift as indicated by the Joint Point software.

In Figure 3, age and BMI adjusted mean reported intakes of fats used as bread spread or for cooking are shown. By far the most common type of fat used as bread spread in northern Sweden in 1986 was a butter-raps seed oil blend (brand name Bregott) with 80% fat until 2004 when different variants holding 80 to 43% fat were introduced (Figure 3 upper panel). Reported intake of the butter-raps seed oil blend spread for bread showed a time trend curve characterized by several changes in the 25 year period, starting with a sharp decrease between 1986 and 1991, followed by an increase to 1994, a slowly progressing decrease until 2005, and thereafter a sharp increase. This pattern was consistent in 10-year age groups (Additional file 2: Figure S2). The opposite was seen for low-fat spread alternatives (Figure 3 upper panel). A linear increase was reported for use of butter and oil for cooking, whereas use of margarine decreased at a similar rate (Figure 3 lower panel and Figure 4.). Largely the increasing trend for use of butter for cooking was similar for men and women and 10-year age groups, although the sharp increase in use of butter for cooking in 2006 was most pronounced among 25–35 year old men and especially 35–45 year old women (Additional file 3: Figure S3).

**Figure 5:**
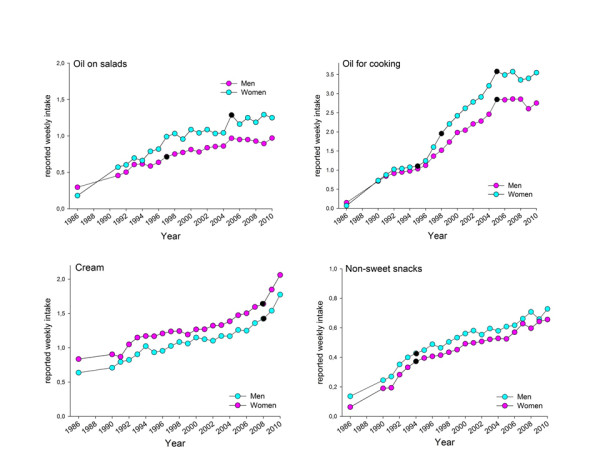
**Reported mean intake of various types of oils used for salad dressing or cooking, cream and cream products, and non-sweet snacks.** Reported weekly mean intake frequencies, adjusted for age and BMI for the period 1986 to 2010 for men and women. Information was not available for 1987 and 1988 since the VIP FFQ was not fully harmonized until 1991. Black dots (●) show years with a trend shift as indicated by the Joint Point software.

Intake of 3% fat milk decreased sharply from 1986 to 1991, and then remained virtually stable until 2008 when a break occurred, and a slightly increasing trend was identified (Figure 4). Use of a medium-fat alternative (1 or 1.5% fat) increased sharply from 1986 to 1991 to a level that was maintained throughout the study period, whereas the low-fat alternative (0.5% fat) was similar from 1986 to 2008 after which a decreasing trend occurred (Figure 4). Use of cream (including regular cream, crème fraiche and sour cream), oils in salad dressings, and non-sweetened snacks (including potato and maize crisps, cheese-flavoured puffed products, pop corn, and peanuts) increased linearly over the 25-year period for men and women (Figure 5). For the other foods identified to contribute to high fat intake, i.e. intake of bacon, hamburgers, sausages, minced meat dishes, steak, stews, pizza and fried potato chips (French fries), reported intakes remained virtually stable over the 25-year period (data not shown).

Among remaining foods or food aggregates, the most striking changes in the 25-year period were sharp, linear decreases in consumption of boiled potato and whole grain crisp bread (data not shown). These changes were balanced by increasing intake of rice and pasta and whole grain soft bread (data not shown). Further, a slight increase in fatty fish intake occurred from around 2000, whereas intake of lean fish was unaltered over the study period (data not shown).

### Alcoholic beverage consumption 1986 to 2010

**Figure 6:**
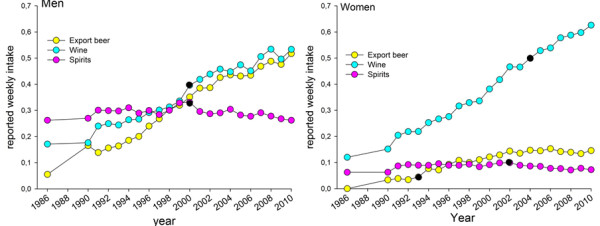
**Reported mean intake of various types of alcohol containing products.** Reported weekly mean intake frequencies, adjusted for age and BMI for the period 1986 to 2010 for men and women. Information was not available for 1987 and 1988 since the VIP FFQ was not fully harmonized until 1991. Black dots (●) show years with a trend shift as indicated by the Joint Point software.

The most striking time trend in intake of alcoholic beverages over the 25-year period was a continuous sharp increase in reported wine consumption (Figure 6). This was especially evident in women. In year 2010 women reported a higher wine intake than men. Over the study period, men also increased their intake of export beer (>3.5 vol% alcohol). Reported intake of spirits (both genders) and export beer in women remained unchanged (Figure 6).

### 25-year trends in two potential markers for diet

**Figure 7:**
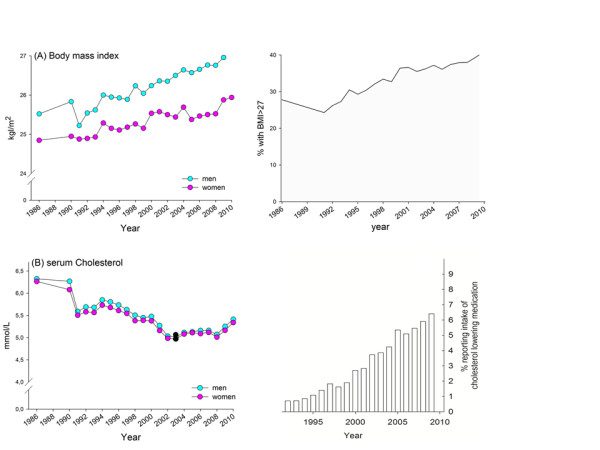
**(A) Mean body mass index (BMI) by study year and proportions with BMI > 27; (B) mean total cholesterol in serum by study year and proportions reporting intake of cholesterol lowering medication by study year.** Means, adjusted for age for the period 1986 to 2010 for men and women. Black dots (●) show years with a trend shift as indicated by the Joint Point software.

Age-adjusted mean BMI and the proportions with a BMI > 27 increased continuously from 1986 to 2010 in the studied Northern Sweden cohort with parallel trend curves for men and women (Figure 7A, upper panel). The increase went from a mean BMI of 25.5 to 27.1 in men and from 24.8 to 25.9 in women. Increases for 10-year age groups are shown in Additional file 4: Figure S4.

In contrast, total cholesterol levels in serum decreased continuously from 1986 to 2004, remained virtually unchanged a few years and then increased after 2007 (Figure 7B). The trend curves were parallel in men and women and in 10-year age groups (Additional file 5: Figure S5. During the 25-year study period use of cholesterol-lowering medication was introduced in the study population. Self-reported use of cholesterol-lowering medication increased linearly from less than 1% of the participants in 1992 to 6.5% in 2010 (Figure 7B, lower right panel). To evaluate if inclusion of participants taking cholesterol-lowering medication affected the trend curves for cholesterol significantly, age adjusted means were compared when these subjects were included or excluded. Age adjusted means for non-medicated subjects only differed in the second decimal digit with no additional effect on the trend breaks compared with when medicated subjects were retained.

### Foods associated with high serum cholesterol levels in men and women in Northern Sweden

We next searched for foods associated with having a high serum cholesterol level by multivariate PLS modelling of energy-providing nutrients/alcohol and foods on cholesterol as a continuous variable in 10-year age groups. In both genders and all age groups models with one significant component were obtained. The explanatory capacities of the models typically were between 4.1 and 2.3% (R^2^ ranged from 0.041 to 0.023). The models remained stable after cross validation (Q^2^ ranged from 0.039 to 0.011).

High consumption of boiled coffee, total fat (E%), saturated fat (grams/day), butter-based spread or butter on bread, margarine for cooking, salted fish, sweet buns and crisp bread rolls, and boiled potato characterised subjects with high versus low levels of cholesterol in serum in six of eight gender/age strata. Other items characterizing subjects with high versus low serum cholesterol levels in at least two gender/age strata were high consumption of total fat (grams/day), butter for cooking, 3% and 1.5% fat milk, medium fat cheese (typically 28% fat), whole grain crisp bread, white bread, sweet fruit soups, and alcohol intake (E%, grams/day, beer and liquor). Some of these associations were gender specific, such as alcohol for men. Mean PLS loading correlations in gender and age group strata are summarized in Additional file 6: Table S1.

## Discussion

The main findings in the present population-based, long-term, follow-up study on food selection, cholesterol levels and BMI were (*i*) a rapid decline in fat intake and concomitant reduction of serum cholesterol levels during the first seven years (1986–1992), but (*ii*) a reversal with pronounced increase in reported saturated and total fat intake during the latest six years (2004–2010) and increasing levels of serum cholesterol levels after 2007. In contrast to serum cholesterol, BMI increased continuously over the 25-year period. In the same period reciprocal trend shifts from carbohydrates to fat were seen. There were also striking linear decreases in consumption of boiled potato and whole grain crisp bread; these were replaced by rice, pasta and whole grain soft bread. A further striking change was a continuous increase in wine consumption, especially in women. Our study design does not allow a causal evaluation of the relationship between the increased fat intake since 2004 and the increased cholesterol values after 2007, although the parallel trends would suggest such a relationship.

### Methodological concerns

The strength of the present study is the population-based sample with a large number of observations and a long follow-up period with sizable variations in the variables explored. Like all studies using reported diet intake methods, more or less severe random and systematic measurement errors and biases may have occurred. Underreporting is a common source of error in both FFQ and interview methods, and this is especially common for recordings of energy, fatty and sweet food in subjects of high BMI [34]. Validation studies are here crucial.

Hence, intake of energy and energy providing nutrients recorded by the presently used FFQ was validated against intakes recorded by repeated 24-hour recalls [28]. The de-attenuated correlation coefficients for total fat intake measured by the two methods were 0.66 for men and 0.59 for women, and underreporting of energy in obese subjects was confirmed when compared to estimated energy need [27]. In the present study, to reduce the effects of random and systematic bias, we (i) used large sample sizes where observations with extreme food intake levels were excluded, i.e. those with very high or low energy intake, (ii) standardized intakes for BMI and reported energy, and (iii) refrained from searching for foods associated with high BMI.

### Comparison with similar intervention studies

The data used for the present study were collected from a population with a unique long-term on-going intervention project. During the 25-year period, the early phase focus was on both individual counselling and intense population-targeting activities. This focus has remained over the years but population activities were most intense in the earlier phase [4,5]. Several other studies have reported results from primary or secondary lifestyle interventions to prevent CVD [37,38]. Even so, the only study that was performed in an overlapping time window with a similarly long observation period, with a similar design of lifestyle intervention, and in a related population, was in northern Finland (North Karelia) [39]. A 35-year evaluation of the North Karelia Project (1972 to 2007) concluded that an intervention can have a major impact on health-related lifestyle and risk factor profiles, and can lead to improved health of the population [39]. Several trends in northern Sweden confirm those found in North Karelia, i.e. reduction in incidence of CVD and smoking prevalence, and the increase of BMI, (39, present study). In both North Karelia and Västerbotten the cholesterol levels fell remarkably during the first phase of the intervention projects, followed by a flatter trend around 2002–2004. However, in contrast to North Karelia, cholesterol tended to increase 2002–2004 and then markedly increased after 2007 in Västerbotten (present study). So far no data showing cholesterol levels in North Karelia after 2007 have been published.

### Support for a link between diet, cholesterol levels and CVD

There is strong support for protective effects of dietary foods/factors in relation to cholesterol levels and coronary heart disease; such positive foods are fish, vegetables, nuts and the “Mediterranean diet” [14,38,40,41]. Harmful dietary factors include trans fatty acids, foods with high glycaemic index, and “Western diet” patterns [14]. For example, the relative risk to develop coronary heart disease is 1.55 (95% CI 1.27–1.83) for subjects eating a “Western diet” [40].

When VIP was launched in 1985 a major focus was to reduce the high cholesterol levels by a change of diet in the direction of a “modified Mediterranean diet” combined with increased physical activity. The diet messages included a reduction of total fat, a shift from saturated to polyunsaturated fatty acids, fewer eggs, but more vegetables, legumes, fruit, fish, and whole grain bread. The decline in cholesterol levels during the first seven years in the present study might reflect the reported changes in food selection, because physical activity did not increase in the period [7]. However, the continuous decrease of cholesterol levels after 1992 and up to 2004 may reflect altered (less saturated) fatty acid profiles in fats used for spreading on bread and cooking. In this period several products that contain canola, olive oil or phytosterols were introduced on the market (the Swedish Dairy Association, personal communication). Furthermore, treatment with blood cholesterol-lowering medication had not been introduced to a significant extent in the initial 7-year period. In Sweden, the use of statins, mainly simvastatin, started after the presentation of the 4S study in 1994 [42]. The validity of self-reported prevalence of cholesterol-lowering medication (a 3.82-fold increase from 1999 to 2009) was supported by a 3.89-fold increase in the number of prescriptions for cholesterol-lowering medication in the region during the same period (data from the National Board of Health and Welfare, http://www.socialstyrelsen.se/statistik/). In Finland the use of lipid-lowering medication was estimated to account for 16% (men) and 7% (women) of the cholesterol reduction between 1982 and 2007, whereas fat reduction and altered fatty acid composition was estimated to account for 65% and 60% of the reduction for men and women, respectively [43]. Similar evaluations, including CVD risk prediction in light of the changing eating habits, cholesterol levels and BMI, is planned in the Västerbotten population, too.

### The unexpected rise of dietary fat intake and cholesterol levels in recent years

Up to 2004, our hypothesis of a stable low level of dietary intake of fats, concurrent with low serum cholesterol levels, compared to initial levels in 1986, was indeed proven correct. Surprisingly, in 2004 a pronounced increase in fat intake was noted and after 2007, cholesterol levels began to increase steadily in both sexes. In parallel, the very-low-carbohydrate and high-fat diets (LCHF [21], i.e. not the traditional reduction of carbohydrates and fat to reduce overall energy intake) became recognized and increasingly popular as a means to lose weight and control blood glucose levels among type-2 diabetics in Sweden. A transition in food selection is now indicated by the present results, local reports of butter shortage in the stores and increased trade of high-fat alternatives (the Swedish Board of Agriculture, http://www.sjv.se). Notably, the increased fat intake was not associated with any reduction or stagnation of the increasing levels of BMI in the northern Sweden population, even though several studies indicate positive effects of LCHF, i.e. weight loss in a short-term perspective [44,45]. However, evidence for weight loss effects beyond six months is lacking [44,45], and long-term safety is controversial, i.e. some studies report adverse health effects [41,46-49] and others do not [50]. Hence, the dietary guidelines of the National Food Administration of Sweden [51], like most other dietary guidelines [14,52], do not support diets with a total fat content exceeding 30–35 E% for these purposes.

Evaluations of 14 randomized trials of statins have concluded that a reduction of LDL cholesterol by 1 mmol/L leads to a 12% reduction in all-cause mortality and a 19% reduction in CHD mortality [53]. Hence, not surprisingly, the promising decline in CHD mortality over the past 20 years has been attributed mainly to healthful changes in blood cholesterol, triglycerides, smoking and hypertension [54]. The decrease in cholesterol alone explained 39% of the mortality reduction [54,55]. Thus, the upward tendency from 2004 and the marked increase of cholesterol after 2007 is a deep concern for both primary and secondary CHD prevention. The long-term deleterious effects of a high blood cholesterol level seem to be neglected in the population and media, and the interest is centred on diets that promise rapid weight loss [44,49]. For the individual standing on the bathroom scale an increase in blood cholesterol may be overlooked, because it will only be detected by measurements at a medical centre.

In conclusion, men and women in northern Sweden decreased their reported intake of total and saturated fat in the first years following the introduction of an intervention programme, but after 2004 fat intake increased, especially saturated fat and butter-based spread for bread and butter for cooking. Supportive opinions in media for high-fat diets seem to have had an impact on consumer behaviour. Initially beneficial and thereafter deleterious changes in blood cholesterol paralleled these trends in food selection, whereas a claimed weight reduction by high-fat diets was not seen in the most recent years. In contrast, BMI increased continuously over the 25-year period. These changes in risk factors may have important effects on primary and secondary prevention of CVD.

## Competing interests

The authors declare that they have no competing interests.

## Authors’ contributions

IJ and AW designed the project. IJ was responsible for data processing and manuscript drafting. BS, KB and GH supported data supply from VIP and MONICA. All authors contributed to manuscript writing, and read and approved the final manuscript.

## Supplementary Material

Additional file 1 **Figure S1.** Mean intake of fat expressed as energy from fat in per cent of total energy intake in age groups by study year. Means, adjusted for BMI for each 10-year age group for men and women for the period 1986 to 2010. Information was not available for 1987 to 1989 since the VIP FFQ was not fully harmonized until 1991, and the reduced FFQ version used in MONICA year 1990 was not acceptable for nutrient estimation. Black dots (●) show years with a trend shift as indicated by the Joint Point software. Unfilled circles indicate 30-year olds with low number in the age group, i.e. <310 subjects. Click here for file

Additional file 2 **Figure S2.** Use of the butter-raps seed oil blend for spreading on bread in age groups by study year. Reported weekly mean intakes, adjusted for BMI for each 10-year age group for men and women for the period 1986 to 2010. Information was not available for 1987 to 1989 since the VIP FFQ was not fully harmonized until 1991. Black dots (●) show years with a trend shift as indicated by the Joint Point software. Unfilled circles indicate 30-year olds with low number in the age group, i.e. <310 subjects. Click here for file

Additional file 3 **Figure S3.** Use of butter for cooking in age groups by study year. Reported weekly mean intakes, adjusted for BMI for each 10-year age group for men and women for the period 1986 to 2010. Information was not available for 1987 to 1989 since the VIP FFQ was not fully harmonized until 1991. Black dots (●) show years with a trend shift as indicated by the Joint Point software. Unfilled circles indicate 30-year olds with low number in the age group, i.e. <310 subjects. Click here for file

Additional file 4 **Figure S4.** Mean BMI in 10-year age groups by study year. Click here for file

Additional file 5 **Figure S5.** Mean serum cholesterol in 10-year age groups by study year. Unfilled circles indicate 30-year olds with low number in the age group, i.e. <310 subjects. Click here for file

Additional file 6 **Table S1.** Foods and energy-providing nutrients significantly associated with having high cholesterol levels. PLS loadings (w*c[1]) for variables in the x-block (see statistics section) where 95% CIs for mean loading plot correlations do not include 0 (zero) in PLS models with cholesterol as a continuous variable. Foods that were significantly associated in at least two age groups per gender are shown. Click here for file

## References

[B1] Wall S Rosén M Nyström L The Swedish mortality pattern: a basis for health planning? Int J Epidemiol 1985 14 285 292 10.1093/ije/14.2.285 4018996

[B2] Tuomilehto J Kuulasmaa K Torppa J WHO MONICA Project: geographic variation in mortality from cardiovascular diseases. Baseline data on selected population characteristics and cardiovascular mortality World Health Stat Q 1987 40 171 184 3617777

[B3] Weinehall L Hellsten G Boman K Hallmans G Prevention of cardiovascular disease in Sweden: the Norsjö community intervention programme - motives, methods and intervention components Scand J Public Health Suppl 2001 56 13 20 11681559

[B4] Brännström I Weinehall L Persson LÅ Wester PO Wall S Changing social patterns of risk factors for cardiovascular disease in a Swedish community intervention project Int J Epidemiol 1993 22 1026 1037 10.1093/ije/22.6.1026 8144283

[B5] Weinehall L Partnership for health. On the role of primary health care in a community intervention programme. Umeå University Medical Dissertations New Series No. 531 1997 Umeå: Umeå University, Department of Epidemiology and Public Health, and Department of Family Medicine

[B6] Stegmayr B Lundberg V Asplund K The events registration and survey procedures in the Northern Sweden MONICA Project Scand J Public Health 2003 31 Suppl 61 9 17 10.1080/14034950310001441 14660242

[B7] Lindahl B Stegmayr B Johansson I Weinehall L Hallmans G Trends in lifestyle 1986-99 in a 25- to 64-year-old population of the Northern Sweden MONICA project Scand J Public Health 2003 31 Suppl. 61 31 37 14660245 10.1080/14034950310001414

[B8] Stegmayr B Eliasson M Rodu B The decline of smoking in northern Sweden Scand J Public Health 2005 33 321 324 discussion 243 10.1080/14034940510032301 16087495

[B9] Eliasson M Janlert U Jansson JH Stegmayr B Time trends in population cholesterol levels 1986–2004: influence of lipid-lowering drugs, obesity, smoking and educational level. The northern Sweden MONICA study J Intern Med 2006 260 551 559 10.1111/j.1365-2796.2006.01730.x 17116006

[B10] Stegmayr B Asplund K Wester PO Trends in incidence, case-fatality rate, and severity of stroke in northern Sweden, 1985–1991 Stroke 1994 25 1738 1745 10.1161/01.STR.25.9.1738 8073452

[B11] Peltonen M Lundberg V Huhtasaari F Asplund K Marked improvement in survival after acute myocardial infarction in middle-aged men but not in women. The Northern Sweden MONICA study 1985–94 J Intern Med 2000 247 579 587 10.1046/j.1365-2796.2000.00644.x 10809997

[B12] Keys A Mienotti A Karvonen MJ Aravanis C Blackburn H Djordjevic BRBS Dontas AS Fidanza F Keys MH Kromhout D Nedeljkovic S Punsar S Seccareccia F Toshima H The diet and 15-year death rate in the Seven Countries Study Am J Epidemiol 1986 124 903 915 3776973 10.1093/oxfordjournals.aje.a114480

[B13] Keys A Seven Countries: a multivariate analysis of death and coronary heart disease 1980 Cambridge: Harvard University Press

[B14] Food and Agriculture Organization of the United Nations (FAO) Fats and fatty acids in human nutrition. Report of an expert consultation 2010 Rome: FAO Food and nutrition paper 91 21812367

[B15] Van Guelpen B Hultdin J Johansson I Hallmans G Stenling R Riboli E Winkvist A Palmqvist R Low folate levels may protect against colorectal cancer Gut 2006 55 1461 1466 10.1136/gut.2005.085480 16638790 PMC1856405

[B16] Nilsson LM Johansson I Lenner P Lindahl B Van Guelpen B Consumption of filtered and boiled coffee and the risk of incident cancer: a prospective cohort study Cancer Causes Control 2010 21 1533 1544 10.1007/s10552-010-9582-x 20512657

[B17] Engström KS Wennberg M Strömberg U Bergdahl IA Hallmans G Jansson JH Lundh T Norberg M Rentschler G Vessby B Skerfving S Broberg K Evaluation of the impact of genetic polymorphisms in glutathione-related genes on the association between methylmercury or n-3 polyunsaturated long chain fatty acids and risk of myocardial infarction: a case-control study Environ Health 2011 10 33 10.1186/1476-069X-10-33 21504558 PMC3103416

[B18] Riboli E Kaaks R The EPIC project: rationale and study design. European prospective investigation into cancer and nutrition Int J Epidemiol 1997 26 Suppl 1 S6 S14 9126529 10.1093/ije/26.suppl_1.s6

[B19] Bingham SA Day NE Luben R Ferrari P Slimani N Norat T Clavel-Chapelon F Kesse E Nieters A Boeing H Tjønneland A Overvad K Martinez C Dorronsoro M Gonzalez CA Key TJ Trichopoulou A Naska A Vineis P Tumino R Krogh V Bueno-de-Mesquita HB Peeters PH Berglund G Hallmans G Lund E Skeie G Kaaks R Riboli E European Prospective Investigation into Cancer and Nutrition: dietary fibre in food and protection against colorectal cancer in the European Prospective Investigation into Cancer and Nutrition (EPIC): an observational study Lancet 2003 361 1496 1501 10.1016/S0140-6736(03)13174-1 12737858

[B20] Nettleton JA McKeown NM Kanoni S Lemaitre RN Hivert MF Ngwa J van Rooij FJ Sonestedt E Wojczynski MK Ye Z Tanaka T Garcia M Anderson JS Follis JL Djousse L Mukamal K Papoutsakis C Mozaffarian D Zillikens MC Bandinelli S Bennett AJ Borecki IB Feitosa MF Ferrucci L Forouhi NG Groves CJ Hallmans G Harris T Hofman A Houston DK Hu FB Johansson I Kritchevsky SB Langenberg C Launer L Liu Y Loos RJ Nalls M Orho-Melander M Renstrom F Rice K Riserus U Rolandsson O Rotter JI Saylor G Sijbrands EJ Sjogren P Smith A Steingrímsdóttir L Uitterlinden AG Wareham NJ Prokopenko I Pankow JS van Duijn CM Florez JC Witteman JC Witteman JC Dupuis J Dedoussis GV Ordovas JM Ingelsson E Cupples LA Siscovick DS Franks PW Meigs JB MAGIC Investigators Interactions of dietary whole-grain intake with fasting glucose- and insulin-related genetic loci in individuals of European descent: a meta-analysis of 14 cohort studies Diabetes Care 2010 33 2684 2691 10.2337/dc10-1150 20693352 PMC2992213

[B21] Malik VS Hu FB Popular weight-loss diets: from evidence to practice Nat Clin Pract Cardiovasc Med 2007 4 34 41 10.1038/ncpcardio0726 17180148

[B22] Hallmans G Agren A Johansson G Johansson A Stegmayr B Jansson JH Lindahl B Rolandsson O Söderberg S Nilsson M Johansson I Weinehall L Cardiovascular disease and diabetes in the Northern Sweden Health and Disease Study Cohort - evaluation of risk factors and their interactions Scand J Public Health Suppl 2003 61 18 24 14660243 10.1080/14034950310001432

[B23] Statistics Sweden Population and Housing Census 1990. Part 7. The planning and processing of the Population and Housing Census 1989 Stockholm: Statistics Sweden

[B24] Weinehall L Hallgren CG Westman G Janlert U Wall S Reduction of selection bias in primary prevention of cardiovascular disease through involvement of primary health care Scand J Prim Health Care 1998 16 171 176 10.1080/028134398750003133 9800231

[B25] Eriksson M Stegmayr B Lundberg V MONICA quality assessments Scand J Public Health Suppl 2003 61 25 30 14660244 10.1080/14034950310001423

[B26] Eriksson M Holmgren L Janlert U Jansson JH Lundblad D Stegmayr B Söderberg S Eliasson M Large improvements in major cardiovascular risk factors in the population of northern Sweden: the MONICA study 1986–2009 J Intern Med 2011 269 219 231 10.1111/j.1365-2796.2010.02312.x 21158982

[B27] Johansson I Hallmans G Wikman Å Biessy C Riboli E Kaaks R Validation and calibration of food frequency questionnaire measurements in the Northern Sweden Health and Disease cohort Public Health Nutr 2002 5 487 496 12003662 10.1079/phn2001315

[B28] Wennberg M Vessby B Johansson I Evaluation of relative intake of fatty acids according to the Northern Sweden FFQ with fatty acid levels in erythrocyte membranes as biomarkers Public Health Nutr 2009 5 1 8 10.1017/S1368980008004503 19144238

[B29] Johansson I Van Guelpen B Hultdin J Johansson M Hallmans G Stattin P Validity of food frequency questionnaire estimated intakes of folate and other B vitamins in a region without folic acid fortification Eur J Clin Nutr 2010 64 905 913 10.1038/ejcn.2010.80 20502473

[B30] Klingberg S Winkvist A Hallmans G Ellegård L Johansson I Evaluation of plant sterol intake estimated with the Northern Sweden Food Frequency Questionnaire Public Health Nutrin press 10.1017/S1368980012003151 PMC10271774 22874465

[B31] Johansson G Wikman Å Åhrén A-M Hallmans G Johansson I Underreporting of energy intake in repeated 24-hour recalls related to gender, age, weight status, day of interview, educational level, reported food intake, smoking habits and area of living Public Health Nutr 2001 4 919 927 11527517 10.1079/phn2001124

[B32] Schofield W Predicting basal metabolic rate, new standards and review of previous work Hum Nutr Clin Nutr 1985 39 Suppl 1 5 41 4044297

[B33] Weinehall L Johnson O Jansson JH Boman K Huhtasaari F Hallmans G Dahlen G Wall S Perceived health modifies the effect of biomedical risk factors in the prediction of acute myocardial infarction. An incident case-control study from northern Sweden J Intern Med 1998 243 99 107 10.1046/j.1365-2796.1998.00201.x 9566637

[B34] Heitmann BL Lissner L Dietary underreporting by obese individuals—is it specific or non-specific? BMJ 1995 311 986 989 10.1136/bmj.311.7011.986 7580640 PMC2550989

[B35] Ståhle L Wold S Multivariate data analysis and experimental design in biomedical research Prog Med Chem 1988 25 291 338 3076969 10.1016/s0079-6468(08)70281-9

[B36] Kim HJ Fay MP Feuer EJ Permutation tests for joinpoint regression with applications to cancer rates Stat Med 2000 19 335 351 10.1002/(SICI)1097-0258(20000215)19:3<335::AID-SIM336>3.0.CO;2-Z 10649300

[B37] Sandvei MS Lindekleiv H Romundstad PR Müller TB Vatten LJ Ingebrigtsen T Njølstad I Mathiesen EB Vik A Risk factors for aneurysmal subarachnoid hemorrhage - BMI and serum lipids: 11-year follow-up of the HUNT and the Tromsø Study in Norway Acta Neurol Scand 2012 125 382 388 21793808 10.1111/j.1600-0404.2011.01578.x

[B38] de Lorgeril M Salen P Martin JL Mediterranean diet, traditional risk factors and the rate of cardiovascular complications after myocardial infarction. Final report of the Lyon Diet Heart Study Circulation 1999 99 779 785 10.1161/01.CIR.99.6.779 9989963

[B39] Vartiainen E Laatikainen T Peltonen M Juolevi A Männistö S Sundvall J Jousilahti P Salomaa V Valsta L Puska P Thirty-five-year trends in cardiovascular risk factors in Finland Int J Epidemiol 2010 39 504 518 10.1093/ije/dyp330 19959603

[B40] Mente A de Koning L Shannon HS Anand SS A Systematic review of the evidence supporting a causal link between dietary factors and coronary heart disease Arch Intern Med 2009 7 659 669 19364995 10.1001/archinternmed.2009.38

[B41] Sjögren P Becker W Warensjö E Olsson E Byberg L Gustafsson IB Karlström B Cederholm T Mediterranean and carbohydrate-restricted diets and mortality among elderly men: a cohort study in Sweden Am J Clin Nutr 2010 92 967 974 10.3945/ajcn.2010.29345 20826627

[B42] No author listed Randomised trial of cholesterol lowering in 4444 patients with coronary heart disease: the Scandinavian Simvastatin Survival Study (4S) Lancet 1994 344 1383 1389 7968073

[B43] Valsta LM Tapanainen H Sundvall J Laatikainen T Männistö S Pietinen P Vartiainen E Explaining the 25-year decline of serum cholesterol by dietary changes and use of lipid-lowering medication in Finland Public Health Nutr 2010 13 932 938 10.1017/S1368980010001126 20513263

[B44] Nordmann AJ Nordmann A Briel M Keller U Yancy WS Brehm BJ Bucher HC Effects of low-carbohydrate vs low-fat diets on weight loss and cardiovascular risk factors: a meta-analysis of randomized controlled trials Arch Intern Med 2006 166 285 293 10.1001/archinte.166.3.285 16476868

[B45] Sacks FM Bray GA Carey VJ Smith SR Ryan DH Anton SD McManus K Champagne CM Bishop LM Laranjo N Leboff MS Rood JC de Jonge L Greenway FL Loria CM Obarzanek E Williamson DA Comparison of weight-loss diets with different compositions of fat, protein, and carbohydrates N Engl J Med 2009 360 859 873 10.1056/NEJMoa0804748 19246357 PMC2763382

[B46] Fung TT van Dam RM Hankinson SE Stampfer M Willett WC Hu FB Low-carbohydrate diets and all-cause and cause-specific mortality: two cohort studies Ann Intern Med 2010 153 289 298 20820038 10.1059/0003-4819-153-5-201009070-00003 PMC2989112

[B47] Lagiou P Sandin S Weiderpass E Lagiou A Mucci L Trichopoulos D Adami HO Low carbohydrate-high protein diet and mortality in a cohort of Swedish women J Intern Med 2007 261 366 374 10.1111/j.1365-2796.2007.01774.x 17391111

[B48] Trichopoulou A Psaltopoulou T Orfanos P Hsieh CC Trichopoulos D Low-carbohydrate-high-protein diet and long-term survival in a general population cohort Eur J Clin Nutr 2007 61 575 581 17136037 10.1038/sj.ejcn.1602557

[B49] Shai I Schwarzfuchs D Henkin Y Shahar DR Witkow S Greenberg I Golan R Fraser D Bolotin A Vardi H Tangi-Rozental O Zuk-Ramot R Sarusi B Brickner D Schwartz Z Sheiner E Marko R Katorza E Thiery J Fiedler GM Blüher M Stumvoll M Stampfer MJ Dietary Intervention Randomized Controlled Trial (DIRECT) Group Weight loss with a low-carbohydrate, Mediterranean, or low-fat diet N Engl J Med 2008 359 229 241 10.1056/NEJMoa0708681 18635428

[B50] Halton TL Willett WC Liu S Manson JE Albert CM Rexrode K Hu FB Low-carbohydrate-diet score and the risk of coronary heart disease in women N Engl J Med 2006 355 1991 2002 10.1056/NEJMoa055317 17093250

[B51] NNR, Nordic Nutrition Recommendations Integrating nutrition and physical activity 2004 Copenhagen: Nordic Council of Ministers

[B52] WHO Diet, nutrition and the prevention of chronic diseases: report of a joint WHO/FAO Expert Consultation. WHO Technical Report Series 916 2003 Geneva: WHO 12768890

[B53] Baigent C Keech A Kearney PM Blackwell L Buck G Pollicino C Kirby A Sourjina T Peto R Collins R Simes R Cholesterol Treatment Trialists' (CTT) Collaborators. Efficacy and safety of cholesterol-lowering treatment: prospective meta-analysis of data from 90,056 participants in 14 randomised trials of statins Lancet 2005 366 1267 1278 16214597 10.1016/S0140-6736(05)67394-1

[B54] Kuulasmaa K Tunstall-Pedoe H Dobson A Fortmann S Sans S Tolonen H Evans A Ferrario M Tuomilehto J Estimation of contribution of changes in classic risk factors to trends in coronary-event rates across the WHO MONICA Project populations Lancet 2000 355 675 687 10.1016/S0140-6736(99)11180-2 10703799

[B55] Björck L Rosengren A Bennett K Lappas G Capewell S Modelling the decreasing coronary heart disease mortality in Sweden between 1986 and 2002 Eur Heart J 2009 30 1046 1056 10.1093/eurheartj/ehn554 19141562

