# Supplementary figures and images for: Associations among 25-year trends in diet, cholesterol and BMI from 140,000 observations in men and women in Northern Sweden
Source: Nutr J. 2012 Jun 11;11:40. doi: 10.1186/1475-2891-11-40 (PMC3489616; doi:10.1186/1475-2891-11-40)

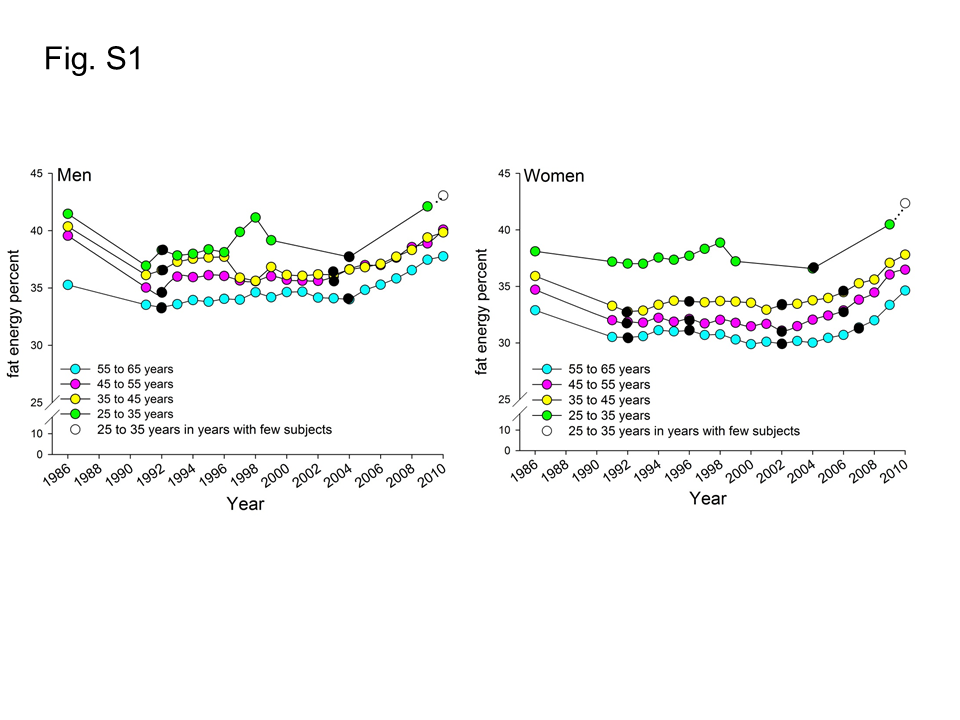

Supplement: Additional file 1 — Figure S1. Mean intake of fat expressed as energy from fat in per cent of total energy intake in age groups by study year. Means, adjusted for BMI for each 10-year age group for men and women for the period 1986 to 2010. Information was not available for 1987 to 1989 since the VIP FFQ was not fully harmonized until 1991, and the reduced FFQ version used in MONICA year 1990 was not acceptable for nutrient estimation. Black dots (●) show years with a trend shift as indicated by the Joint Point software. Unfilled circles indicate 30-year olds with low number in the age group, i.e. <310 subjects. [file 1475-2891-11-40-S1.tiff]

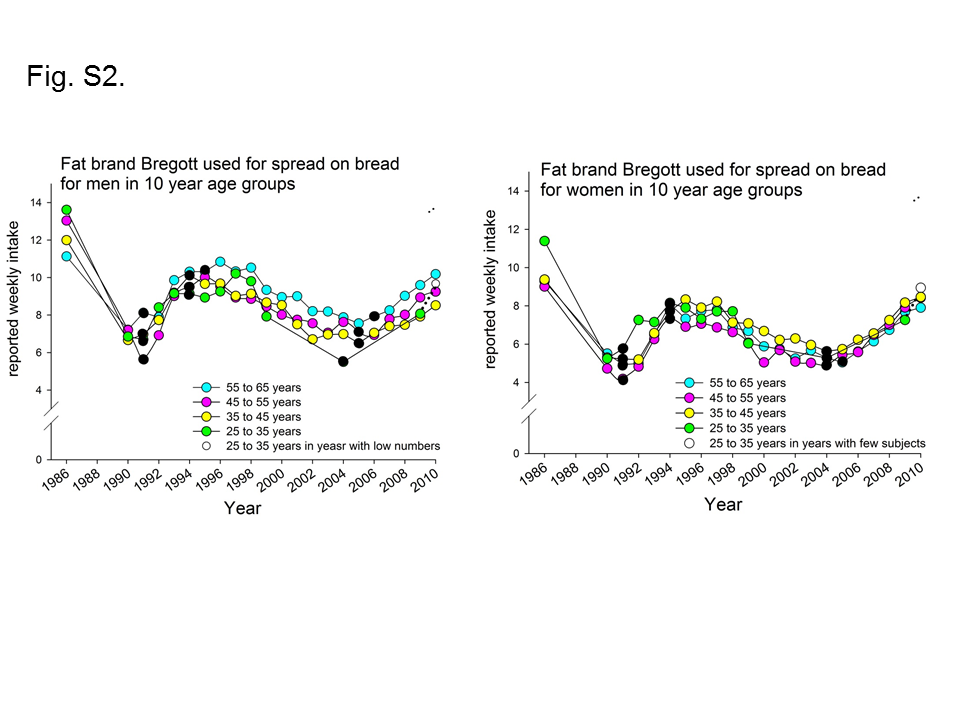

Supplement: Additional file 2 — Figure S2. Use of the butter-raps seed oil blend for spreading on bread in age groups by study year. Reported weekly mean intakes, adjusted for BMI for each 10-year age group for men and women for the period 1986 to 2010. Information was not available for 1987 to 1989 since the VIP FFQ was not fully harmonized until 1991. Black dots (●) show years with a trend shift as indicated by the Joint Point software. Unfilled circles indicate 30-year olds with low number in the age group, i.e. <310 subjects. [file 1475-2891-11-40-S2.tiff]

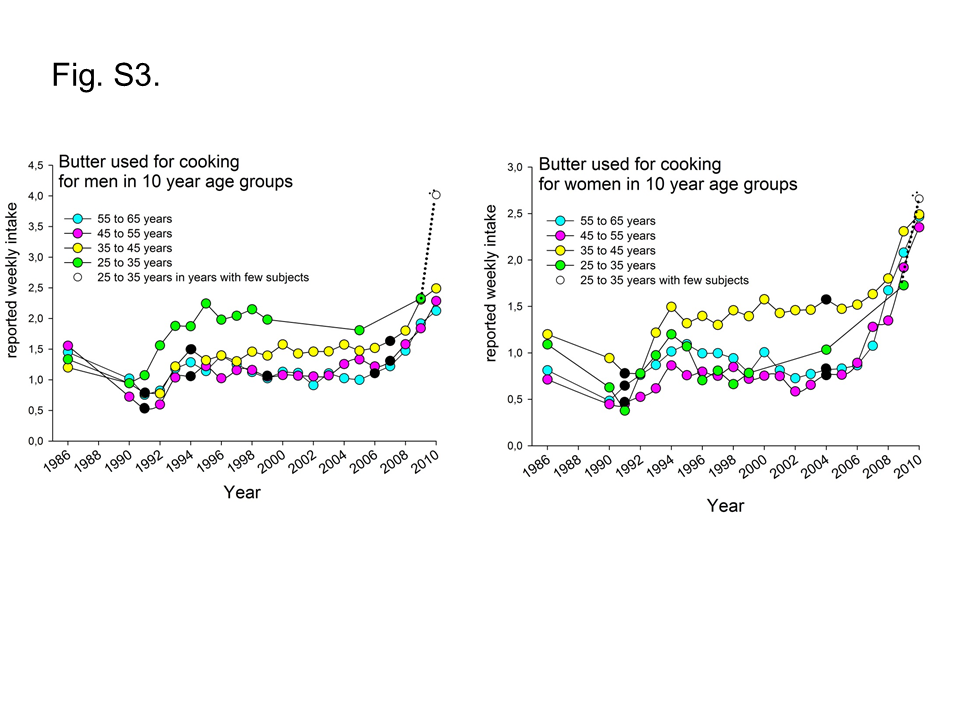

Supplement: Additional file 3 — Figure S3. Use of butter for cooking in age groups by study year. Reported weekly mean intakes, adjusted for BMI for each 10-year age group for men and women for the period 1986 to 2010. Information was not available for 1987 to 1989 since the VIP FFQ was not fully harmonized until 1991. Black dots (●) show years with a trend shift as indicated by the Joint Point software. Unfilled circles indicate 30-year olds with low number in the age group, i.e. <310 subjects. [file 1475-2891-11-40-S3.tiff]

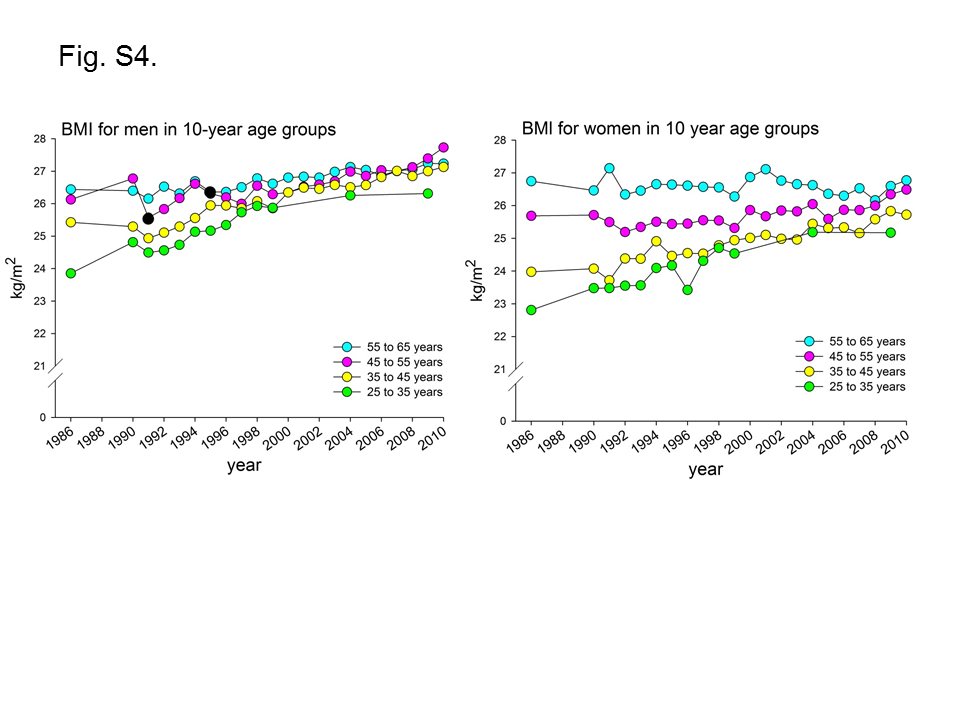

Supplement: Additional file 4 — Figure S4. Mean BMI in 10-year age groups by study year. [file 1475-2891-11-40-S4.tiff]

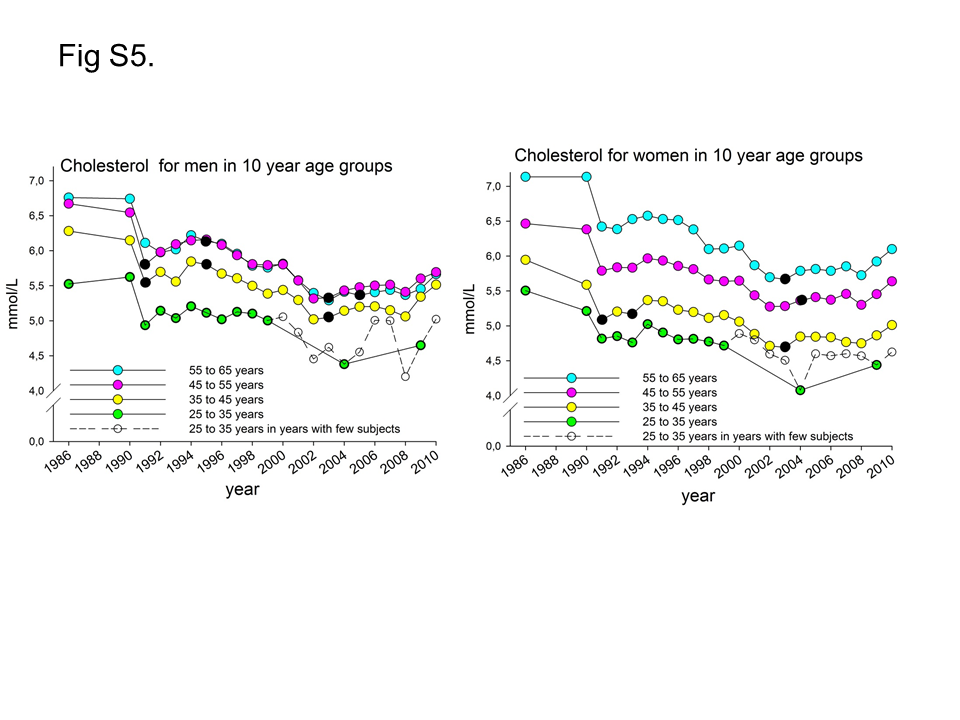

Supplement: Additional file 5 — Figure S5. Mean serum cholesterol in 10-year age groups by study year. Unfilled circles indicate 30-year olds with low number in the age group, i.e. <310 subjects. [file 1475-2891-11-40-S5.tiff]
