# Supplementary material for: Associations among 25-year trends in diet, cholesterol and BMI from 140,000 observations in men and women in Northern Sweden
Source: Nutr J. 2012 Jun 11;11:40. doi: 10.1186/1475-2891-11-40 (PMC3489616; doi:10.1186/1475-2891-11-40)
Supplement: Additional file 6 — Table S1. Foods and energy-providing nutrients significantly associated with having high cholesterol levels. PLS loadings (w*c[1]) for variables in the x-block (see statistics section) where 95% CIs for mean loading plot correlations do not include 0 (zero) in PLS models with cholesterol as a continuous variable. Foods that were significantly associated in at least two age groups per gender are shown. [file 1475-2891-11-40-S6.docx]

**Supplementary Table 1.** **Foods and energy-providing nutrients significantly associated with having high cholesterol levels.** PLS loadings (w*c[1]) for variables in the x-block (see statistics section) where 95% CIs for mean loading plot correlations do not include 0 (zero) in PLS models with cholesterol as a continuous variable. Foods that were significantly associated in at least two age groups per gender are shown.

|  | PLS loading correlations when modelling  energy providing nutrients or food intake on serum cholesterol levels | | | | | | | | |
| --- | --- | --- | --- | --- | --- | --- | --- | --- | --- |
|  | Women | | | |  | Men | | | |
|  | 25-35 years | 35-45 years | 45-45 years | 55-65 years |  | 25-35 years | 35-45 years | 45-45 years | 55-65 years |
| Boiled, unfiltered coffee | 0.310 | 0.392 | 0.347 | 0.331 |  | 0.372 | 0.337 | 0.258 | 0.323 |
| Fat (E%) | 0.270 | 0.067 | 0.135 | 0.194 |  | 0.213 | 0.227 | 0.144 | - |
| Saturated fat (g/day) | 0.170 | 0.100 | 0.115 | 0.197 |  | 0.148 | 0.106 | 0.089 | 0.138 |
| Fat (gram/day) | 0.100 | - | - | 0.103 |  | 0.142 | 0.064 | - | 0.066 |
| Alcohol (E%) | - | - | - | - |  | - | 0.072 | 0.126 | - |
| Alcohol (gram/day) | - | - | - | - |  | - | - | 0.110 | 0.066 |
| Butter-raps seed oil as spread on bread | 0.163 | 0.1176 | 0.1062 | 0.161 |  | 0.177 | 0.143 | 0.156 | 0.175 |
| Butter as spread on bread | 0.107 | 0.076 | 0.0884 | 0.144 |  | 0.092 | - | 0.085 | - |
| Butter for cooking | 0.080 | - | - | 0.096 |  | - | - | - | - |
| Margarine for cooking | 0.100 | 0.169 | 0.085 | 0.082 |  | 0.223 | 0.148 | 0.135 | 0.108 |
| Milk (3% fat, unfermented) | 0.271 | 0.115 | 0.156 | 0.183 |  | - | - | - | - |
| Milk (1.5% fat, unfermented) | - | - | - | - |  | 0.107 | 0.099 | 0.091 | 0.150 |
| Hard cheese (medium fat, 28%) | 0.131 | - | - | 0.080 |  | - | - | - | - |
| Salted fish (herring) | 0.160 | 0.063 | 0.062 | 0.065 |  | - | 0.099 | 0.065 | 0.075 |
| Bacon | - | - | - | - |  | 0.097 | 0.078 | 0.080 | - |
| Meat stew | - | - | - | - |  | 0.178 | 0.072 | - | - |
| Buns, rolls | 0.132 | 0.163 | 0.161 | 0.232 |  | - | 0.059 | - | 0.149 |
| Whole grain, high fibre crisp bread | - | 0.160 | 0.117 | - |  | - | 0.165 | 0.157 | 0.097 |
| White, soft bread | - | 0.112 | 0.102 | - |  | - | - | - | - |
| Potato, boiled or baked | - | 0.229 | 0.1455 | 0.082 |  | 0.244 | 0.254 | 0.154 | 0.114 |
| Fried potatoes | - | 0.056 | - | 0.051 |  | - | 0.079 | 0.067 | - |
| Rosemary or juice soup, fruit cream | - | 0.083 | 0.077 | - |  | - | - | - | - |
| Spirits | - | - | - | - |  | 0.103 | 0.101 | 0.161 | 0.099 |
| Beer with ≤2.25 vol% alcohol | - | - | - | - |  | - | 0.074 | 0.091 | 0.081 |
| Beer with 2.25-3.5 vol% alcohol | - | - | - | - |  | - | 0.091 | 0.103 | 0.097 |
